# Discriminating severe seasonal allergic rhinitis. Results from a large nation-wide database

**DOI:** 10.1371/journal.pone.0207290

**Published:** 2018-11-28

**Authors:** Davide Caimmi, Nour Baiz, Shreosi Sanyal, Soutrik Banerjee, Pascal Demoly, Isabella Annesi-Maesano

**Affiliations:** 1 Unité d’allergologie, Département de Pneumologie et Addictologie, University Hospital of Montpellier, CHU de Montpellier, France; 2 Epidemiology of Allergic and Respiratory Diseases Department (EPAR), Sorbonne Université, INSERM, Pierre Louis Institute of Epidemiology and Public Health, Saint-Antoine Medical School, Paris, France; Telethon Institute for Child Health Research, AUSTRALIA

## Abstract

Allergic rhinitis (AR) is a chronic disease affecting a large amount of the population. To optimize treatment and disease management, it is crucial to detect patients suffering from severe forms. Several tools have been used to classify patients according to severity: standardized questionnaires, visual analogue scales (VAS) and cluster analysis. The aim of this study was to evaluate the best method to stratify patients suffering from seasonal AR and to propose cut-offs to identify severe forms of the disease. In a multicenter French study (PollinAir), patients suffering from seasonal AR were assessed by a physician that completed a 17 items questionnaire and answered a self-assessment VAS. Five methods were evaluated to stratify patients according to AR severity: k-means clustering, agglomerative hierarchical clustering, Allergic Rhinitis Physician Score (ARPhyS), total symptoms score (TSS-17), and VAS. Fisher linear, quadratic discriminant analysis, non-parametric kernel density estimation methods were used to evaluate miss-classification of the patients and cross-validation was used to assess the validity of each scale. 28,109 patients were categorized into “mild”, “moderate”, and “severe”, through the 5 different methods. The best discrimination was offered by the ARPhyS scale. With the ARPhyS scale, cut-offs at a score of 8–9 for mild to moderate and of 11–12 for moderate to severe symptoms were found. Score reliability was also acceptable (Cronbach’s *α* coefficient: 0.626) for the ARPhyS scale, and excellent for the TSS-17 (0.864).

The ARPhyS scale seems the best method to target patients with severe seasonal AR. In the present study, we highlighted optimal discrimination cut-offs. This tool could be implemented in daily practice to identify severe patients that need a specialized intervention.

## Introduction

Allergic rhinitis (AR) affects up to 50% of some populations, especially in “westernized” countries and its prevalence, in France, tripled over the last 25 years and is around 31% [1,2]. Current guidelines try to categorize patients by differentiating between those suffering from “mild” symptoms and those with “moderate to severe” forms of rhinitis [3–5]. There is not a unanimous way to define patients as simply “severe”, even though it seems important to highlight this specific group, because of the consistent burden associated to these patients, in terms of increased morbidity and therefore direct healthcare and indirect socio-economic cost [6,7].

ARIA guidelines look for duration and types of symptoms reported to the physician, and the new allergy diary App for smartphones questions patients on how they feel, through a visual analogue scale (VAS) [4,8]. Therefore, if on one hand, current classification is based on physician’s appreciation of the disease as presented by the patient, on the other hand, it highlights the importance of how the patient feels, regardless the items proposed by the guideline. The real goal, both in the “classical” ARIA classification and in the ARIA App for patients’ self-evaluation, is to assess the control of the disease, regardless its severity. In fact, AR control implies that patients do not present bothersome symptoms when exposed to allergens, while severe forms characterize patients who are not able to control their symptoms even if an appropriate high-dose treatment is prescribed and their compliance is good.

Several scores have been previously validated to assess AR control, such as the CARAT, the RCAT, the ARCT, and the VAS (both on a pencil-and-paper tool and through smartphones) [2,9]. At the same time, some scores, including symptoms scores and VAS, have been tested or even validated to assess patients’ severity and categorize patients [10–14]. A few authors proposed on the other hand to assess severe AR, by analyzing patients in clusters, and stratify them, based on the severity of their symptoms [15–17]. Therefore, in literature, severe forms may be identified through physicians’ questionnaire, self-assessment methods, or by analyzing the results of published cluster analysis. Besides identifying the best tool to stratify patients, it is debated whether physician’s or patient’s assessment would serve as the best guide to classify the patient’s severity.

The aim of the present paper was therefore to assess the best method to stratify patients suffering from seasonal AR and to then propose cut-offs able to determine which patients suffer from severe forms of rhinitis.

## Materials and methods

### Study design

In a multicenter French study, 36,397 adult patients with a previous medical diagnosis of seasonal AR and consulting a physician were included. All patients were consulting either a general practitioner or an ENT, or an allergist, or a dermatologist, or a pulmonologist. A total of 8,143 doctors distributed over the whole French territory participated to the study. The PollinAir study was approved in France in 2005. The approval by an ethic committee was declared as not applicable at that time. Instead of the study was approved by the National Committee for Information Management on medical research (Comité Consultatif sur le Traitement de l’Information en Matière de Recherche dans le domaine de la santé) and by the National Commission on informatics and health (CNIL, Commission Nationale Informatique et Liberté). Information was provided to included subjects or to their caregivers through a written document. Informed written consent to participate in the survey was obtained for all patients by the physicians. The CNIL approved in 2016 that all data acquired prior to 2016, without the previous need of an authorization of an Ethic Committee, could still be exploited. The survey and its methodology have been described in detail elsewhere [10,18].

### Collected data

Each doctor interviewed the patients after confirming the previous medical diagnosis of seasonal AR through a clinical visit, and answered 17 questions for each included patient; each question focused on one item, and the physician was supposed to rate every symptom from 0 to 4 in a 5-point Likert scale (0: absent, 1: mild, 2: moderate, 3: severe, and 4: very severe). Evaluated symptoms were: nasal congestion, nasal obstruction, rhinorrhea, nasal itching, sneezing, headache, tiredness, loss of appetite, irritability, lacrimation, eye itching, painful throat, cough, itching throat, earache, alteration of daily activity, and sleep alteration. Other collected data included age, gender, location (rural / urban), disease onset (years before), duration of episode (days), reported history of asthma, conjunctivitis, atopic dermatitis, food allergy, or hives, results of skin-prick tests (SPTs) to respiratory allergens (positive / negative), positivity of serum specific IgE to respiratory allergens (positive / negative), previous or concomitant allergen immunotherapy (yes / no), and region (center, east, north-west, Paris agglomeration, south-east, south-west and west). On the day of the visit, patients completed a Visual Analogue Scale (VAS) on a paper, indicating, on a 10-cm line, how severe they believed their rhinitis was (“how bothersome are your allergic rhinitis symptoms?”).

### Data and statistical analysis

Five approaches to classify patients according to AR severity were assessed:

1. **K-means clustering (KMC)** [19] was used as unsupervised classification on standardized variables to categorize AR patients. A group of three clusters were then selected for further analyses.
2. **Agglomerative hierarchical clustering (AHC)** [20] was used as unsupervised classification on standardized variables to categorize AR patients. A group of three clusters were then selected for further analyses.
3. **Allergic Rhinitis Physician Score** (**ARPhyS**), previously described as “Global Symptomatic Score (GSS-20)” [10,18] was calculated, based on five physician-diagnosed symptoms. These symptoms were assessed by each doctor during the interview with the patients. To each nasal (nasal obstruction, rhinorrhea, sneezes and nasal pruritus) and ocular symptom (ocular pruritus), doctors attributed a severity score ranging from 0 to 4, as described above. This score could therefore possibly range from a minimum of 0 to a maximum of 20 points. The score was categorized into three terciles.
4. **Total Symptoms Score (TSS-17)** [21,22], which is the global score resulting from adding up the evaluation of the 17 items rated by physicians for each included patient. This score could therefore possibly range from a minimum of 0 to a maximum of 68 points. The score was categorized into three terciles.
5. **Visual Analogue Scale (VAS)** [11,12], which is a global self-assessment wellness score reported by each patient, and ranging from 0 (= no discomfort) to 100 (= maximal discomfort). The score was categorized into three terciles.

Classification in three groups had been chosen to follow current guidelines on AR severity and their adaptation [3,23,24]. For validation, K-means algorithm and agglomerative hierarchical clustering models were carried out 10 times by the leave-one-out method to ensure stability and repeatability of the models.

Classical statistical methods were used for the analysis [19–21,25–27]. Discrimination analyses was conducted with Fisher linear and quadratic discriminant analysis, along with non-parametric kernel density estimation methods [28] and allowed to evaluate miss-classification of the patients among the three categories on each of the five scales. Cross-validation check was used to assess the validity of each scale [29]. Reliability of the ARPhyS scale and of the TSS-17 were evaluated through Cronbach’s alpha coefficient. At last, Cohen’s κ coefficient was computed to study the degree of agreement between the five scales.

All analyses were performed using SAS version 9.4 (SAS Institute Inc, Cary, NC, USA). All *p-values* <0.05 were considered statistically significant.

## Results

### Patients’ stratification

**Fig 1:**
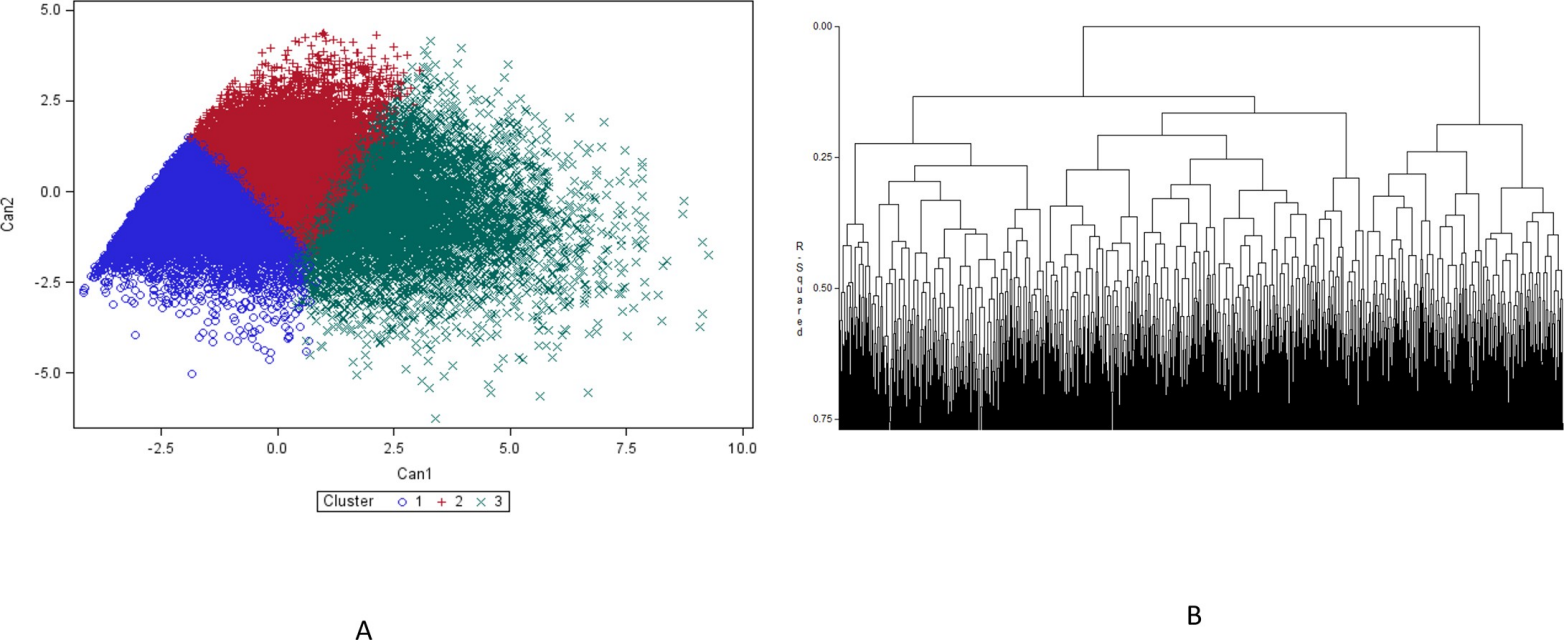
Cluster analysis through two methods (all variables standardized). (A) K-means clustering, non-hierarchical clustering approach; k = 3 (Cluster 1 = ‘mild’ allergic rhinitis; Cluster 2 = ‘moderate’ allergic rhinitis; Cluster 3 = ‘severe’ allergic rhinitis). (B) Agglomerating hierarchical clustering dendrogram, with y-axis that shows the R^2^ as the distance measure (R^2^ = 16.5%).

Out of the 36,397 subjects initially included in the trial, 8,288 were excluded from further analysis, because of missing data. The other 28,109 patients were then categorized in three classes, of “mild”, “moderate”, and “severe” AR, as shown in Table 1. Stratification by cluster analysis is shown in Fig 1. There were 10,617 patients in the mild, 9446 patients in the moderate and 8046 patients in the severe category based on the ARPhyS scale (Table 1).

**Table 1:** Distribution of mild, moderate, and severe patients, based on the five approaches used in the study.

	Mild		Moderate		Severe	
	Included subjects, n (%)	Cumulated frequency (n)	Included subjects (n)	Cumulated frequency (n)	Included subjects (n)	Cumulated frequency (n)
		Cumulated Percentage (%)		Cumulated Percentage (%)		Cumulated Percentage (%)
KMC	11358 (40.41)	11358	10163 (36.16)	21521	6588 (23.44)	28109
		40.41		76.56		100.00
AHC	8021 (28.54)	8021	12403 (44.12)	20424	7685 (27.34)	28109
		25.54		72.66		100.00
ARPhyS	10617 (37.77)	10617	9446 (33.60)	20063	8046 (28.62)	28109
		37.77		71.38		100.00
TSS-17	9579 (34.08)	9579	9177 (32.65)	18756	9353 (33.27)	28109
		34.08		66.73		100.00
VAS	9247 (32.90)	9247	9689 (34.47)	18936	9173 (32.63)	28109
		32.90		67.37		100.00

The difference in terciles group is due to the TIES = Low option (default): ties are assigned to lower categories.

CV: cross-validation; KD: kernel density; KMC: k-means clustering; AHC: agglomerative hierarchical clustering; ARPhyS: Allergic Rhinitis Physician

Score; TSS-17: Total Symptom Score with 17 items; VAS: Visual Analogue Scale (0–100).

### Discrimination and cross-validation

When considering all the five approaches, the best discrimination was offered by the ARPhyS scale, followed by the KMC, and then by the TSS-17 and by the AHC, while the VAS produced the worst results (Table 2). For validation, K-means algorithm and agglomerative hierarchical clustering models were carried out 10 times by the leave-one-out method to ensure stability and repeatability of the models. These methods showed 95.6 and 94.8% % repeatability. The ArPhyS scale showed the best results in terms of error rates and cross-validation error rates, as highlighted in Table 2. Based on the ARPhyS stratification in mild, moderate and severe symptoms, the characteristics of the included patients are shown in Table 3. The duration of the rhinitis episode did not have a statistically significant impact on the ARPHyS scale, despite the large sample size. The proportion of rural population was significantly higher in the severe category compared to the mild category by approximately 3%; no statistically significant difference was noted in gender distributions across the categories (Table 3). The number of patients presenting with a history of conjunctivitis, asthma, atopic dermatitis, food allergy, and hives, and with positive SPT or specific IgE or with a previous or concomitant allergen immunotherapy significantly increased with severity.

**Table 2:** Overall error (misclassification) rates and cross-validation error rates according to the approaches and the different discrimination methods.

Discrimination methods	KMC		AHC		ARPhyS		TSS-17		VAS	
	Error rates (%)	CV error rates (%)	Error rates (%)	CV error rates (%)	Error rates (%)	CV error rates (%)	Error rates (%)	CV error rates (%)	Error rates (%)	CV error rates (%)
Linear	4.30	4.40	22.39	22.48	3.08	3.18	8.31	8.36	43.93	44.06
Quadratic	10.96	11.20	23.36	23.58	2.89	3.06	9.34	9.48	45.43	45.95
KD with equal bandwidth	0.04	11.50	0.67	15.62	0.00	7.92	0.00	11.43	1.85	48.53
KD with unequal bandwidth	0.99	15.43	5.62	23.97	0.12	8.11	2.43	16.18	8.07	50.87

CV: cross-validation; KD: kernel density; KMC: k-means clustering; AHC: agglomerative hierarchical clustering; ARPhyS: Allergic Rhinitis Physician Score; TSS-17: Total Symptom Score with 17 items; VAS: Visual Analogue Scale (0–100).

Smoothing parameter for hierarchical clusters was varied from 0.4 to 1.0 for sensitivity analysis. A moderate smoothing bandwidth (0.8) showed the best results for all analyses. Linear discrimination was better than quadratic discrimination for all analysis. ARPhyS, TSS-17 and VAS are divided into tertiles; KMC and AHC are grouped into 3 clusters.

**Table 3:** Characteristics of the patients according to allergic rhinitis severity as assessed with the ARPhyS Scale.

Characteristics	Mild (N = 10,617)		Moderate (N = 9,446)		Severe (N = 8,046)		*p-value*^1^
	Mean	SE	Mean	SE	Mean	SE	
Age (years)	35.12	0.15	34.30	0.15	33.32	0.16	< 0.001
Onset (years ago)	6.85	0.07	7.38	0.07	7.91	0.08	< 0.001
Duration of episode (days)	19.64	0.22	19.23	0.23	19.70	0.26	0.500
Loss of appetite	0.22	0.00	0.42	0.01	0.69	0.01	< 0.001
Nasal congestion	2.18	0.01	2.63	0.01	3.02	0.01	< 0.001
Daily activity disturbed	0.80	0.01	1.16	0.01	1.54	0.01	< 0.001
Sneezing	1.68	0.01	2.52	0.01	3.17	0.01	< 0.001
Tiredness	0.83	0.01	1.20	0.01	1.63	0.01	< 0.001
Painful throat	0.55	0.01	0.76	0.01	1.11	0.01	< 0.001
Irritability	0.46	0.01	0.78	0.01	1.16	0.01	< 0.001
Lacrimation	0.95	0.01	1.54	0.01	2.27	0.01	< 0.001
Earache	0.14	0.01	0.22	0.01	0.38	0.01	< 0.001
Nasal obstruction	0.94	0.01	1.58	0.01	2.25	0.01	< 0.001
Ears/Throat itching	0.45	0.01	0.82	0.01	1.33	0.01	< 0.001
Nasal itching	1.12	0.01	1.97	0.01	2.79	0.01	< 0.001
Ocular itching	0.60	0.01	1.32	0.01	2.29	0.01	< 0.001
Rhinorrhoea	1.87	0.01	2.56	0.01	3.13	0.01	< 0.001
Sleep disturbed	0.87	0.01	1.25	0.01	1.64	0.01	< 0.001
Headache	0.60	0.01	0.88	0.01	1.20	0.01	< 0.001
Cough	0.73	0.01	1.00	0.01	1.27	0.01	< 0.001
	**N (%)**	**SE**	**N, %**	**SE**	**N, %**	**SE**	
Female	5,550 (52.27)	0.48	4,933 (52.22)	0.51	4,191 (52.09)	0.56	0.970
Rural	4,332 (40.80)	0.48	4,013 (42.48)	0.51	3,532 (43.90)	0.55	< 0.001
History of conjunctivitis	6,349 (59.80)	0.49	6,762 (71.59)	0.48	6,679 (83.01)	0.43	< 0.001
History of asthma	2,227 (20.98)	0.41	2,402 (25.43)	0.47	2,404 (29.88)	0.53	< 0.001
History of atopic dermatitis	1,472 (13.86)	0.35	1,554 (16.45)	0.40	1,521 (18.91)	0.46	< 0.001
History of food allergy	647 (6.09)	0.24	662 (7.01)	0.28	691 (8.59)	0.33	< 0.001
History of hives	1,766 (16.63)	0.38	1,780 (18.84)	0.42	1,761 (21.89)	0.48	< 0.001
Positive SPT	2,064 (19.44)	0.44	1,962 (20.77)	0.47	2,052 (25.52)	0.55	< 0.001
Positive specific IgE	1,042 (9.81)	0.32	1,104 (11.69)	0.37	1,153 (14.33)	0.43	< 0.001
Previous or concomitant AIT	813 (7.66)	0.26	660 (6.99)	0.27	721 (8.96)	0.33	< 0.001
Region - Centre - East - North-west - Paris agglomeration - South-east - South-west - West	755 (7.11) 1,232 (11.60) 1,518 (14.30) 2,020 (19.03) 2,068 (19.48) 1,615 (15.21) 1,409 (13.27)	0.25 0.31 0.34 0.38 0.38 0.35 0.33	706 (7.47) 1,210 (12.81) 1,317 (13.94) 1,643 (17.39) 1,975 (20.91) 1,410 (14.93) 1,185 (12.54)	0.27 0.34 0.36 0.39 0.42 0.37 0.34	585 (7.27) 1,068 (13.27) 1,022 (12.70) 1,442 (17.92) 1,766 (21.95) 1,245 (15.47) 918 (11.41)	0.29 0.38 0.37 0.43 0.46 0.40 0.35	< 0.001

SPT: skin prick tests; AIT: Allergen Immunotherapy; SE: Standard Error.

^1^ Kruskal-Wallis test for continuous or ordinal variables, and χ^2^-test for categorical variables.

### Reliability, agreement and stratification

Score reliability, assessed through Cronbach’s *α* coefficient, was acceptable (0.626, computed on the original raw scores) for the ARPhyS scale, and excellent for the TSS-17 (0.864). Maximum variability was observed in the first canonical component direction explaining 99.8% of the total variability for the ARPhyS scale. As for agreement between scores, VAS showed the lowest agreement if compared with all the other scores, as shown in Table 4.

**Table 4:** Cohen’s *κ* matrix showing agreement between the different methods of classification.

	KMC	AHC	ARPhyS	TSS-17	VAS
KMC	1	0.49	0.41	0.67	0.28
AHC	0.49	1	0.33	0.54	0.24
ARPhyS	0.41	0.33	1	0.45	0.25
TSS-17	0.67	0.54	0.45	1	0.32
VAS	0.28	0.24	0.25	0.32	1

KMC: k-means clustering; AHC: agglomerative hierarchical clustering; ARPhyS: Allergic

Rhinitis Physician Score; TSS-17: Total Symptom Score with 17 items; VAS: Visual Analogue Scale

(0–100). ARPhyS, TSS-17 and VAS are divided into terciles; KMC and AHC are grouped into 3 clusters.

In order to choose cut-offs able to properly stratify patients, based on the ARPhyS scale, we identified those values that would be best associated to equivalent previously highlighted terciles: we propose therefore cut-offs at a score of 8–9 for mild to moderate symptoms and of 11–12 for moderate to severe symptoms. To summarize, patients were classified as presenting with “mild” symptoms if they scored 0 to 8 with the ARPhyS scale; they had “moderate” symptoms if they scored 9 to 11; they should be considered as “severe” whenever they scored 12 to 20 (Table 5).

**Table 5:** The ARPhyS score, with cut-offs level to identify patient’s severity.

ARPhyS							
Please, rate the severity of each of the following symptoms as presented in this moment by your patient:							
							Score
A	Nasal obstruction	0	1	2	3	4	
		Absent	Mild	Moderate	Severe	Very severe	
B	Rhinorrhea	0	1	2	3	4	
		Absent	Mild	Moderate	Severe	Very severe	
C	Sneezes	0	1	2	3	4	
		Absent	Mild	Moderate	Severe	Very severe	
D	Nasal pruritus	0	1	2	3	4	
		Absent	Mild	Moderate	Severe	Very severe	
E	Ocular pruritus	0	1	2	3	4	
		Absent	Mild	Moderate	Severe	Very severe	
						Total Score	
If total score is 8 or less, then your patient presents **mild** symptoms								
If total score is between 9 and 11, then your patient presents **moderate** symptoms							
If total score is 12 or more, then your patient presents **severe** symptoms							

### Missing data analysis

When analyzing data from the 8,288 patients excluded from the cohort, because of missing data, no significant statistical difference was highlighted when considering sex, age, history of conjunctivitis, asthma, atopic dermatitis, food allergy, hives, and positive SPT. Significant difference was on the contrary pointed out when evaluating other items: proportion of rural population included in the analysis was significantly higher than those who were not included due to missing data (43.4% vs. 39.9%; *p* < 0.001); same considerations as for positive specific IgE (11.9% vs. 11.0%; *p* = 0.036); at last, the included population underwent previous or concomitant allergen immunotherapy almost significantly more than the excluded group (7.9% vs. 7.3%; *p* = 0.048).

## Discussion

When dealing with patients suffering from allergic rhinitis, physicians should be able to easily detect those presenting severe forms. In fact, severe AR patients may often suffer from comorbidities, may need more drugs to control their symptoms, and may present an impaired quality of life, besides being at risk for an increased loss of productivity and absenteeism [2]. The cost of AR is therefore elevated when considering severe patients; in a recent study by Colás et al., the authors estimated that the cost of severe AR is of 2.965,28 € per year, including direct and indirect costs [7]. Therefore, it seems important to be able to promptly recognize severe forms to provide patients proper efficient treatments.

In recent years, cluster analysis has become more and more common to identify subgroups of patients: it consists in applying unsupervised statistical methods to a population with a wide distribution of related symptoms, and then identifying possible homogeneous phenotypes, with minimum overlap between each other [15]. In a work by Burte et al., the authors highlight three different clusters of rhinitis (allergic and non-allergic), from a population of 983 adults, but they do not differentiate them based on severity [17]. In a work by Bousquet PJ et al., on the contrary, the authors identified clusters of severe AR, in a population of 990 patients, and then compared them with the ARIA classification [15]. They found that, in *real-life*, physicians prescribe a therapy, with no regard to nasal symptoms severity [15], and therefore current guidelines and proposed cluster do not help general practitioners in stratifying the severity of patients presenting with AR. We identified three clusters, through two different methods, in a population of 28,109 patients. Clusters showed no overlap between each other. After evaluation through Fisher linear and quadratic discriminant analysis, and non-parametric kernel density estimation methods, we found that cluster analysis does not provide the best results in terms of discrimination, error rate and cross-validation, if compared to other assessed methods (Table 2).

Visual Analogue Scales, on the other hand, have been used for several diseases in recent years. They have been tested and validated for AR, even on smartphones screens [9]. This approach, advised by the novel ARIA guidelines, is useful to assess symptoms control and quality of life impairment, but also severity in patients suffering from AR [8,12]. In fact, a recent paper by Del Cuvillo et al. showed in a population of 3,572 patients that a VAS greater than 7 cm is a reliable score to identify severe patients (Negative Predictive Value, NPV, of a VAS above 7: 98.6%; Positive Predictive Value, PPV, at 7: 20.4%) [12]. A previous paper by Bousquet PJ et al., on a cohort of 3,052 patients, proposed a 5-cm cut-off for mild forms, while moderate to severe patients were to be considered for a VAS of over 6 cm (with a NPV of 56.5% and a PPV of 94.3%) [11]. The two papers found two different cut-offs, but in the study by Bousquet PJ et al., the authors only differentiated “mild” patients from “moderate/severe”, while the study by del Cuvillo categorized patients into three severity groups (mild, moderate, and severe). On the other hand, in a paper by Rouve et al., it seemed that categorizing AR severity in patients through VAS brought to an exaggerated inclusion in the severe group [10]. In the present paper, we found that VAS proved the worst results in terms of discrimination and cross-validation (Table 2), and the least agreement in terms of results if compared with the other 4 tested methods (Table 4). Considering our findings from a very large cohort of patients, we may speculate that VAS is a useful tool for diagnosis and assessment of disease control, for both patients and physicians, but not the best tool for classifying patients according to severity by physicians. Another possible explanation of the discrepancy between our results and those from del Cuvillo and Bousquet PJ is that we only included patients suffering from seasonal AR, while the previous authors did not use such selection criterion.

In contrast with the ARIA guidelines, we found that the duration of the disease does not have a significant impact on the severity of symptoms, and such result confirms what had been previously stated by Bousquet PJ et al. [15]. Rather than differentiating the disease between intermittent and persistent, attention should be focused on nasal and ocular symptoms, based on our findings. In a study by Valero et al., the authors evaluated a TSS-4 to stratify AR patients, based on the clinical items proposed by the ARIA guidelines [14], and in a following study the authors validated the TSS-4 as a tool to assess severe forms [13]. Through these papers, Valero et al. underlined that total symptoms scores seem to be practical methods for physicians to target severe patients, following current ARIA guidelines. Based on such considerations, in our study, even though the ARPHysS scale showed a Cronbach’s alpha-coefficient inferior to the TSS-17, we chose the first questionnaire because, besides being superior as a discriminating method, it is also quicker and easier to use in everyday clinical practice. Also, the ARPhyS scale (Table 5) allowed us to identify tertiles that maximally correlated in the first canonical dimension and therefore to propose simple cut-offs to categorize AR into “mild”, “moderate”, and “severe”. [11]. On the other hand, we really wanted to highlight the importance of identifying severe patients, since mild ones usually do not even consult their general practitioner for rhinitis symptoms, while severe may need a specialized approach in order to control their symptoms and comorbidities.

A possible limit of the study is that patients visiting physicians were only evaluated during spring and summer seasons, which might limit the generalizability of our results to patients visiting during autumn and winter seasons. However, the large sample size provides robustness in the results and we were able to propose a practical tool for physicians, which is fast and obtained from a *real-life* study.

The ARIA classification of AR severity is useful, especially to differentiate mild patients from the others. Several tools have been developed for physicians to assess severity and control in recent years. Such tools need to be easy-to-use and efficient, and, so far, only a few of them have been validated. Through the present study, we evaluated two different tools to assess AR severity: one composed by 17 questions and the other one, the ARPhyS scale, by 5 questions. When comparing our tool to the other tested methods, we found that the ARPhyS scale is the best in terms of discrimination and cross-validation. Also, it is an easy tool for physicians and we found some cut-off values able to differentiate mild patients from moderate, and from severe ones. Such tool could therefore be implemented in daily practice to identify severe patients that need a specialized intervention or anyway a more important therapeutic treatment.
